# Follow Your Heart: How Is Willingness to Pay Formed under Multiple Anchors?

**DOI:** 10.3389/fpsyg.2017.02269

**Published:** 2017-12-22

**Authors:** Chien-Huang Lin, Ming Chen

**Affiliations:** Department of Business Administration, National Central University, Taoyuan, Taiwan

**Keywords:** assimilation effect, contrast effect, external reference price, internal reference price, multiple anchoring, willingness to pay

## Abstract

In sales, a common promotional tactic is to supplement a required purchase (i.e., a focal product) by offering a free or discounted product (i.e., a supplementary product). The present research examines the contextual factors driving consumer evaluations of the supplementary product after the promotion has been terminated. Two experiments are used to demonstrate that consumers use multiple anchors to determine the value of a supplementary product. Consumers use other types of price information, such as the internal reference price (IRP), promotional price, and original price of the supplementary product, as anchors to adjust their willingness to pay. Among the multiple anchors, the consumer’s IRP is not only the crucial anchor to estimate the willingness to pay but also the criterion to determine whether other price information can serve as anchors. Price information, such as the promotional and original price of the supplementary product, which is higher (lower) than the IRP, will increase (decrease) the willingness to pay. However, these anchors are only employed when the price information is considered to be plausible. Assimilation and contrast effects occur when the IRP is used by consumers as a criterion to judge the reasonableness of other anchors. When the external price information belongs (does not belong) to consumers’ distribution of IRP, assimilation (contrast) effects occur, and consumers will regard the external reference price (ERP) to be a plausible (implausible) price. Limitations and future avenues for research are also discussed.

## Introduction

Offering a product for different prices is a common strategy companies use to promote products and attract consumers. There is a wealth of research examining how to use diverse pricing strategies in varying circumstances. Among the various pricing strategies, two fairly common strategies are offering a product for a discounted price or offering it for free with the purchase of another product (Palmeira and Srivastava, 2013). For example, a clothing store may sell a belt for $5, or it may offer the belt for free with the purchase of a $100 item. Consider these two situations: you purchase the $100 item and receive the belt for free or purchase the $100 item and receive the belt for $5. If you want to purchase a similar belt after the promotion ends, what is the first thing that comes to mind when you consider your willingness to pay? Will your willingness to pay be different if your friend received the belt for free versus for the discounted price? Will other factors, such as the original price of the belt, influence your willingness to pay? The present research addresses these issues.

As Neslin (2002) notes, “conditional promotions” (e.g., purchasing a focal product and receiving the supplementary product for free or for a discounted price) are used to boost short-term sales. Sellers and researchers have been examining the impact of conditional promotions on consumers’ willingness to pay for a supplementary product. According to Kamins et al. (2009), offering any product in a bundle as “free” makes consumers less willing to pay for that product when unbundled and sold individually. Similarly, Raghubir (2004) argue that the low cost of the supplementary product makes consumers less willing to pay for it as a stand-alone product after the conditional promotion has been terminated. These studies all suggest that offering a product for free lowers its perceived value so the consumer is less willing to pay for it. However, other results suggest that free products have a positive effect on consumers and draw more attention (Nunes and Park, 2003; Chandran and Morwitz, 2006). For example, Palmeira and Srivastava (2013) proposed that offering a supplementary product for free instead of at a discounted price renders consumers more willing to pay a higher price for the product once the conditional promotion has been withdrawn.

Given the conflicting points of view, this research focuses on the contextual factors of how consumers determine their willingness to pay for a supplementary product after different types of temporary promotions are terminated. This study predicts that consumers use multiple anchors to determine their willingness to pay for a supplementary product [e.g., internal reference price (IRP), the price of the focal product, the original price of the supplementary product, and the promotional price of the supplementary product]. In addition, the present study also explores how these multiple anchors jointly influence consumers’ judgment (e.g., the relationship between these anchors).

## Theoretical Background

Three bodies of literature are most relevant to this research: the *reference price* literature, which examines why IRP is a crucial anchor; the *anchoring point* literature, which examines the contextual factors that drive consumers to estimate and modulate their willingness to pay for a product; and the *assimilation-contrast theory* literature, which examines how consumers choose and determine whether varying price information can be employed as anchors when determining their willingness to pay for a product. This section discusses each of these literature streams and explains how they are used to support the hypotheses.

### Reference Price

There is a wealth of research providing insights into reference prices, how they can be measured or modeled, and how they affect consumer purchase behavior (Mazumdar et al., 2005). The effects of reference price on consumer choice have been recognized; moreover, the concept of a reference point has been extended to other stimuli such as price promotions (Lattin and Bucklin, 1989), product quality (Hardie et al., 1993), and expected price (Wenner, 2015).

Mazumdar et al. (2005) defined a reference price as a price prediction that is shaped by a consumer’s past purchasing experience and current purchasing environment. According to their definition, there are two types of reference price, internal and external, that are jointly used in consumers’ purchase decisions. The IRPs may be stored in consumers’ memory (Winer, 1986), but external reference prices (ERPs) are clearly visible in stores (Kopalle and Lindsey-Mullikin, 2003). Due to the vast amount of information provided by the purchase environment, consumers have to determine which pieces of external information they can use to assimilate and integrate into their IRP (Mazumdar et al., 2005), which influences their purchase behaviors or evaluations (e.g., Urbany et al., 1988; Lichtenstein and Bearden, 1989). Moreover, many researchers have focused on what influences a consumer’s reference price. According to Briesch et al. (1997), the most important factor in a consumer’s IRP is the previously observed price for the product. Furthermore, adaptation-level theory suggests that the IRP can be influenced by other cues, such as previously acquired information or expectations for the product’s quality based on other products in the same category or on the specific brand (Grewal et al., 1998). Rajendran and Tellis (1994) also found that several individual characteristics (e.g., the strength of brand preference) can moderate consumers’ relative weighting of the two types of reference price when making decisions. For example, the temporal component of reference (IRP) is more efficient for consumers who have a strong brand preference than the other contextual components (ERP); in contrast, consumers who sample a wide variety of brands may pay more attention to the contextual component than to the temporal component. In addition, the external information in the store environment also significantly affects the consumer’s purchasing decision. A consumer’s expectation for a product’s price may differ across various stores depending on the level of service, the assortment of products offered in the store, and the type of store (Mazumdar et al., 2005). For example, a bottle of wine sold in a luxury hotel may be regarded more favorably than the same bottle sold in a discount wine store for the same price. The information provided by a price promotion is a typical type of ERP; for example, one of the most prevalent formats for an ERP is “Compare at $X” (Lichtenstein et al., 1991). Aside from the “Compare at $X” format, there are other types of contextual information.

The promotional methods discussed in the present research (i.e., purchasing a focal product and gaining a supplementary product for free or for a discounted price) are also typical ERPs used in store environments. In the situation discussed in the research, all ERPs are under the condition of a temporary promotional period. However, this study focuses on consumers’ willingness to pay for the supplementary product when the promotion is terminated, whether the external price information in the promotion is considered by consumers, and whether they integrate this information to form a new IRP that may influence their willingness to pay.

### Multiple Anchoring Judgment

As discussed above, contextual information, especially price information such as ERP, can significantly influence a consumer’s judgment about a product. Recent literature suggests that contextual information can also affect a consumer’s willingness to pay (Adaval and Wyer, 2011). Palmeira and Srivastava (2013) proposed that an anchoring process can explain a consumer’s willingness to pay for a supplementary product. They suggested that the price of the focal product is used as an anchor when the supplementary product is offered for free, while the discounted price of the supplementary product is used as an anchor when it is offered at a discount.

As research on the anchoring effect suggests, price anchors not only provide a reference for price estimations but also activate thoughts that are consistent with the anchor (Tversky and Kahneman, 1974; Strack and Mussweiler, 1997; Adaval and Wyer, 2011). Tversky and Kahneman (1974) proposed that once an anchor is set, people adjust away from it to get to their final answer. However, the adjustment is insufficient, resulting in a final guess that is closer to the anchor than it would be otherwise (Tversky and Kahneman, 1974; Epley and Gilovich, 2001; Mussweiler and Strack, 2004). It has been argued that during the anchoring and adjusting procedure (Tversky and Kahneman, 1974; Yadav, 1994), the selected anchor serves as an initial point to which upward or downward adjustments are made in response to new information encountered. It has been argued in several prior studies on insufficient adjustment that people adjust their evaluation from starting points they generate themselves even though they know the anchors are incorrect but only close to the right answer (Epley and Gilovich, 2001, 2006). For example, Americans who may not know exactly when Abraham Lincoln was elected president of the United States can estimate the date by adjusting from the date of the Emancipation Proclamation in 1862 or the date he was murdered in 1865 – in other words dates which are close to the correct answer. Thus, we propose that consumers evaluate the product, starting at their IRP, and then making upward or downward adjustments when external price information is taken into consideration. However, the adjustment of external price information (e.g., ERP) on consumers’ IRP is influenced by the plausibility of the ERP (Urbany et al., 1988) and the difference between the ERP and the actual selling price (Kopalle and Lindsey-Mullikin, 2003). Building on previous research, this study argues that when the external price information (e.g., the price of the focal product when the supplementary product is offered for free, the original price of the supplementary product, and the discounted price of the supplementary product when it is offered at a discount) in the promotion is considered by consumers as plausible price information, the ERP will integrate into their IRP by increasing or decreasing the original IRP to the new IRP and influence consumers’ willingness to pay when the promotion is terminated. When the ERP is higher (lower) than consumers’ original IRP, it will increase (increase) consumers’ new IRP, which increases (decreases) consumers’ willingness to pay after the promotion ends. Conversely, when consumers do not regard the ERP as plausible price information, this ERP cannot influence consumers’ IRP or their willingness to pay after the promotion ends. Thus, the following hypotheses are proposed:

(1) When the ERP is regarded as plausible price information by consumers, it can increase (decrease) consumers’ willingness to pay for the supplementary product after the promotion ends if it is higher (lower) than consumers’ IRP.
(1) When the ERP is not regarded as plausible price information by consumers, it cannot influence consumers’ willingness to pay for the supplementary product after the promotion ends.

### Criteria for Judging the Plausibility of Contextual Price Information

**FIGURE 1:**
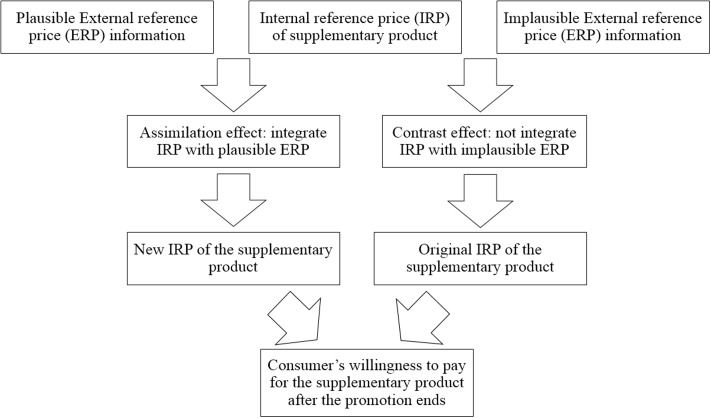
The complete conceptual framework.

Due to the significant influence of contextual information, it is necessary to explore the underlying criteria used by consumers to judge the validity of information. Building on previous research about consumer price evaluations (Sherif and Hovland, 1961; Meyers-Levy and Sternthal, 1993; Kopalle and Lindsey-Mullikin, 2003; Cunha and Shulman, 2011), this study argues that the assimilation and contrast effects can be used to describe how consumers evaluate abundant contextual information and integrate it into their IRP and that these effects further influence consumers’ willingness to pay after the promotion ends. Herr (1989) suggested that the feature overlap between the context and the target product dictates whether assimilation or contrast effects occur. As argued above, consumers use multiple types of contextual price information as anchors to estimate their willingness to pay for a supplementary product. We propose that consumers employ their IRP, which is the initial anchor, as a criterion to determine whether they will apply external price information. According to assimilation-contrast theory (Sherif and Hovland, 1964), consumers have a distribution of prices that they consider to be acceptable. Contextual price information will be assimilated only if judged to fall within that distribution (Lichtenstein and Bearden, 1989). The types of contextual price information discussed include the price of the focal product, the promotional price of the supplementary product, and the original price of the supplementary product. The complete conceptual framework is shown in **Figure 1**.

Along with the assimilation and contrast effects, social judgment theory, as proposed by Sherif et al. (1965), is also helpful to explain how consumers judge the contextual price information. They argued that people’s attitude is an amalgam of three latitudes: acceptance, neutrality (non-commitment), and rejection. The latitude of acceptance is the range of information that a person considers as reasonable or worthy of consideration; the latitude of rejection is the range of information that a person considers as unreasonable or objectionable; and the latitude of neutrality (non-commitment) which lies in the middle of these opposites is the range of information that a person considers as neither acceptable nor questionable (Sherif and Hovland, 1961; Sherif et al., 1965; Griffin, 2011). Similarly, when consumers evaluate the contextual price information, they use their IRP as one of the criteria to judge which latitude the contextual price information belongs to. When the contextual price information belongs to the latitude of neutrality or rejection, it does not become a plausible anchor because the information is considered irrelevant or unreasonable. Contextual price information can be a useful anchor only when it falls within the latitude of acceptance. Thus, the following hypotheses are proposed:

(1) Consumers use their IRP as a criterion to judge whether the ERP is plausible contextual price information or not.

## Experiment 1

The purpose of Experiment 1 was to explore how consumers determine the plausibility of the promotional price of the supplementary product and how they use the plausible or implausible price information to estimate their willingness to pay for the supplementary product after the promotion ends.

### Method

As proposed, whether the price information belongs to the distribution of consumers’ IRP is the criterion that determines the plausibility of the price information. However, measuring the distribution of consumers’ IRP is difficult. Prior research did not suggest an accurate range of this distribution: approximately 0.75 times the price variability of the product (Kalyanaram and Little, 1994); within +4% of the regular price of the brand (Kalwani and Yim, 1992); or a probabilistic range (Han et al., 2001). Thus, we manipulated the plausibility of the promotional price of the supplementary product by manipulating the gap between the IRP and the promotional price of the supplementary product. The promotional price, which has a large (small) gap between the IRP and the promotional price of the supplementary product, can be regarded by consumers as implausible (plausible) price information. Thus, a pre-test for consumers’ IRP of the supplementary product was conducted based on the following question: How much are you willing to pay for this product? Headphones and vacuum cups were used as the supplementary products. These items were selected because of their price, the headphones being relatively high in price which provided enough price range to manipulate the plausibility of the promotional price, that is implausible for the headphones but plausible for the vacuum cup. A total of 15 undergraduate students participated in the pre-test. They were shown six different pictures of headphones and vacuum cups with no brand logo and subsequently provided the price that they were willing to pay. After the analyses, a pair of headphones (*M*_IRP_ = NT$953.13) and a vacuum cup (*M*_IRP_ = NT$293.87) were selected as the supplementary product for this experiment.

Following Kalyanaram and Little (1994), we manipulated the plausible promotional price by 0.25 times the consumer’s IRP. A manipulation check was also conducted to ensure the success of the manipulation. The IRP for the headphones, as assessed from the pre-test results was NT$953.13, thus “receive these wonderful headphones for NT$150/NT$250” was selected as indicative of the condition where the consumer would regard the price as implausible because of the large gap between the IRP and the promotional price (NT$150/NT$250 vs. NT$953.13). Conversely, NT$150/NT$250 was closer to the IRP of the vacuum cup (*M*_IRP_ = NT$293.87). Thus, “receive this wonderful vacuum cup for NT$150/NT$250” was selected as the plausible condition with only a small gap between the IRP and the promotional price. To examine whether a higher promotional price can increase consumers’ willingness to pay, two promotional prices (NT$150 vs. NT$250) were employed in each condition.

### Samples and Procedure

The sample in this study was selected for convenience. A total of 120 undergraduate students (54% males; *M*_age_ = 21.71) at a large public university in Taiwan participated in this 2 (price of the suplementary product: high vs. low) × 2 (plausibility of the promotional price: implausible vs. plausible) between-subjects study in exchange for course credit. Participants were randomly assigned one of four conditions and shown an ad: “Buy an NT$10,000 smartphone and receive these wonderful headphones (this wonderful vacuum cup) for NT$150 (NT$250).” For each condition, the ad included the same picture of the smartphone and the headphones (or vacuum cup) without brand information.

### Measures

The participants were asked to indicate their willingness to pay for the headphones (or vacuum cup) after the promotion was terminated. As a manipulation check for the plausibility of the promotional price, the participants were also asked to indicate responses to statements on a seven-point scale (1 = strongly disagree, 7 = strongly agree): “The promotional price is implausible after termination of the promotion.”

### Results and Discussion

Two participants did not respond to all of the study questions. Thus, the analyses are based on 118 participants. The manipulation check indicated that participants in the implausible condition (headphones) (*M* = 5.19, *SD* = 1.43) reported that the promotion price was significantly more plausible than did the participants in the plausible condition (vacuum cup) [*M* = 3.64, *SD* = 1.40; *F*(1,116) = 35.02, *p* < 0.001].

**FIGURE 2:**
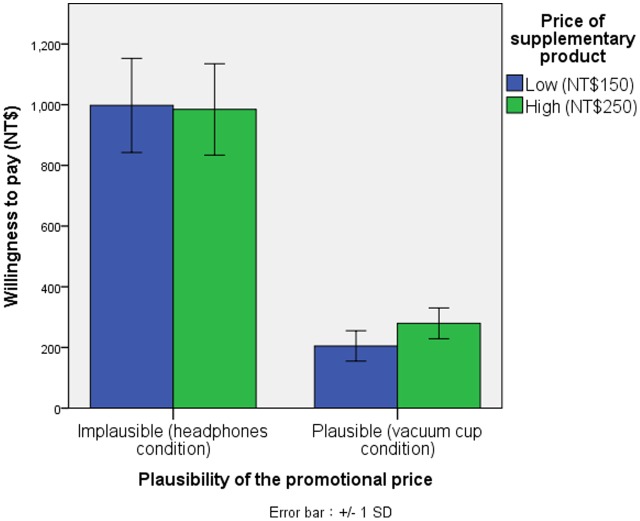
Interaction between the promotional price and its plausibility of the supplementary product.

A 2 × 2 ANOVA of consumers’ willingness to pay revealed significant main effects for the plausibility of the promotional price [*F*(1,114) = 1276.24, *p* < 0.001, η^2^ = 0.912] but insignificant main effects for the price of the supplementary product [*F*(1,114) = 2.14, NS]. A significant two-way interaction between the two factors [*F*(1,114) = 4.37, *p* < 0.05, η^2^ = 0.030] was observed. Planned contrasts did not reveal a significant difference in the willingness to pay across high price conditions (NT$250) (*M* = NT$997.63, *SD* = 155.00) and low price (NT$150) conditions [*M* = NT$984.45, *SD* = 150.68; *F*(1,114) < 1, NS] when the promotional price was considered to be an implausible price (headphones condition). In contrast, when the promotional price was considered to be a plausible price (vacuum cup condition), there was a significant difference in willingness to pay across the high price (NT$250) (*M* = NT$279.45, *SD* = 50.65) and low price (NT$150) conditions [*M* = NT$204.97, *SD* = 50.16; *F*(1,114) = 6.31, *p* < 0.05, **Table 1** and **Figure 2**].

**Table 1:** Willingness to pay for the headphones (or vacuum cup) after termination of the promotion.

Plausibility of the promotional price	Promotional price ofsupplementary product	Mean
Implausible	Low (NT$150)	NT$997.63
(headphones condition)	High (NT$250)	NT$984.45
Plausible	Low (NT$150)	NT$204.97
(vacuum cup condition)	High (NT$250)	NT$279.45


Experiment 1 provides strong support for the proposed multiple anchoring judgment theory: the same promotional price with different gaps between the promotional price and the IRP of a supplementary product caused varied willingness to pay for the supplementary product after the promotion ends. Palmeira and Srivastava (2013) argued that consumers only use this price as an anchor to estimate their willingness to pay if a supplementary product is offered for a discounted price; the willingness to pay for the vacuum cup and the willingness to pay for the headphones should not differ because they were both offered for the same discounted price. However, the results of this experiment indicated a difference and indicated a crucial role of the IRP in estimating willingness to pay after the promotion ended. The pre-test suggested that the IRP of the headphones was approximately NT$953.13. When they were offered for NT$150 or NT$250, a large gap between the promotional price and the IRP was observed. Due to the contrast effect, the participants did not use the promotional price as plausible price information to integrate their IRP. Consumers may consider this price as a very special price only for a brief promotional period. Thus, when the promotion was terminated, they continued to use their original IRP for the headphones as an anchor to estimate their willingness to pay, which produced no change in their willingness to pay despite the promotional price of NT$150 or NT$250. Conversely, when the vacuum cup was offered for NT$150 or NT$250 (both closer to the IRP NT$293.87), the assimilation effect dictated that the participants regarded the promotional price as plausible price information. Thus, the promotional price of the vacuum cup can be integrated with consumers’ original IRP to create a new IRP. The new IRP can be used as an anchor for consumers to estimate their willingness to pay for the supplementary product after the promotion ends. As a result, consumers who observed the vacuum cup offered for NT$150 had a lower willingness to pay than consumers who observed the vacuum cup offered for NT$250. Although the price of a focal product was also considered as an anchor, the price of the focal product was the same in all conditions in Experiment 1. Thus, this finding does not explain the reason for this differentiation.

## Experiment 2

The purpose of Experiment 2 was to provide further evidence for our theory by examining how consumers use additional ERP information—the original price of the supplementary product—to estimate their willingness to pay for the supplementary product after the promotion ends.

### Method

Similar to the manipulation employed in Experiment 1, the plausibility of the original price of the supplementary product was manipulated by manipulating the gap between the IRP and the original price of the supplementary product. The original price, which has a large (small) gap between the IRP and the original price of the supplementary product, can be regarded by consumers as implausible (plausible) price information. Mugs and vacuum cups were used as the supplementary products because of the relatively low price of the mug which allowed us to easily manipulate the original price to be implausible for the mug but plausible for the vacuum cup. In addition, another product was used to explore increasing the validity of the effect. A pre-test completed by 15 undergraduate students was conducted to test the IRP of the supplementary product. They were shown six different pictures of mugs without any price or brand information. The participants then reported how much they would be willing to pay for the product. After the analyses, a mug (*M*_IRP_ = NT$93.60) was chosen as the supplementary product for Experiment 2. In addition, the vacuum cup (*M*_IRP_ = NT$293.87) employed in Experiment 1 was also employed in this study.

The IRP of the mug as assessed from the pre-test was NT$93.60. Similar to the manipulation employed in Experiment 1, “receive a beautiful mug originally priced at NT$300/NT$400” was selected as indicative of the implausible original price condition, with a large gap between the IRP and the original price for the supplementary product. Conversely, because NT$300/NT$400 was closer to the IRP of the vacuum cup (*M*_IRP_ = NT$293.87), “receive a beautiful vacuum cup originally priced at NT$300/NT$400” was selected as indicative of the plausible original price condition, with a small gap between the IRP and the original price. To examine whether a higher original price can increase consumers’ willingness to pay, two prices were used in each condition (NT$300 vs. NT$400).

### Samples and Procedure

The sample in this study was selected for convenience. A total of 120 undergraduate students (35% males; *M*_age_ = 20.86) at a large public university in Taiwan were randomly assigned to one of four conditions in a 2 (original price of the supplementary product: low vs. high) × 2 (plausibility of original price of the supplementary product: implausible vs. plausible) between-subjects study in exchange for course credit. The participants were shown the following ad: “Spend over NT$1000 and receive a beautiful mug (vacuum cup) originally priced at NT$300 (NT$400) for free!” All ads included pictures of the mug (the vacuum cup).

### Measures

The participants reported their willingness to pay for the mug (the vacuum cup) after the promotion was terminated and the plausibility of the original price (1 = extremely implausible, 7 = extremely plausible) as a manipulation check.

### Results and Discussion

Three participants did not respond to all of the study questions and were removed from the analyses. Compared with participants in the plausible condition (vacuum cup condition), the manipulation check indicated that participants in the implausible condition (mug condition) (*M* = 1.90, *SD* = 0.94) reported that the original price of the mug was significantly less plausible [*M* = 5.02, *SD* = 1.07; *F*(1,115) = 281.16, *p* < 0.001].

**FIGURE 3:**
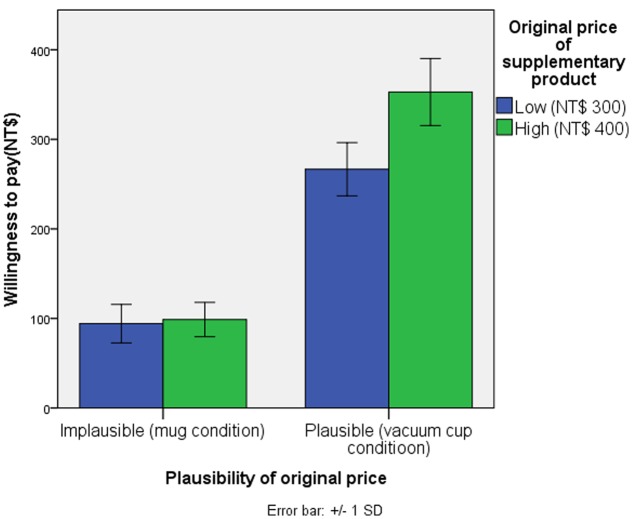
Interaction between the original price and its plausibility of the supplementary product.

A 2 × 2 ANOVA of consumers’ willingness to pay revealed main effects for both the amount of the original price [*F*(1,113) = 77.42, *p* < 0.01, η^2^ = 0.039] and the plausibility of the original price of the supplementary product on the participants’ willingness to pay [*F*(1,113) = 1704.38, *p* < 0.001, η^2^ = 0.86]. A significant interaction between the amount and the reasonableness of the original price [*F*(1,113) = 62.35, *p* < 0.001, η^2^ = 0.031] was observed. When the original price of the supplementary product was implausible (mug condition), no significant differences in willingness to pay were observed between high original conditions (NT$400, *M* = NT$98.83, *SD* = 19.17) and low original price conditions [NT$300, *M* = NT$94.17, *SD* = 21.45, *F*(1,113) < 1, NS]. However, if the original price of the supplementary product was reasonable (vacuum cup condition), the participants were willing to pay a higher price for the supplementary product with a high original price (NT$400, *M* = NT$ 352.83, *SD* = 37.39) than for the supplementary product with a low original price [NT$300, *M* = NT$ 266.61, *SD* = 29.77; *F*(1,113) = 138.12, *p* < 0.001, **Table 2** and **Figure 3**].

**Table 2:** Willingness to pay for the mug (or vacuum cup) after the termination of the promotion.

Plausibility of the original price	Promotional price ofsupplementary product	Mean
Implausible	Low (NT$300)	NT$94.17
(mug condition)	High (NT$400)	NT$98.83
Plausible	Low (NT$300)	NT$ 266.61
(vacuum cup condition)	High (NT$400)	NT$ 352.83


Experiment 2 provides additional evidence for the proposed multiple anchoring judgment theory. Although the original price of a supplementary product is a crucial anchor for consumers when determining their willingness to pay, the relationship between the original price and the IRP of a supplementary product can influence whether the original price is a useful or meaningful anchor. If the gap between the original price and the IRP of a supplementary product is excessive, the contrast effect causes consumers to regard the original price as implausible; thus, they would not integrate it with their IRP to create a new IRP. Consequently, consumers’ original IRP would be used as an anchor to estimate their willingness to pay when the promotion was terminated. Hence, neither a higher nor a lower original price can influence consumers’ willingness to pay for the supplementary product. However, when the gap between the original price and the IRP is small, the assimilation effect can cause consumers to consider the original price as plausible external price information and integrate it with their original IRP to create a new IRP for the supplementary product. Thus, a higher original price would increase consumers’ new IRP for the supplementary product and increase their willingness to pay relative to a lower original price. The original price of a supplementary product can be used to integrate consumers’ original IRP into a new IRP and estimate consumers’ willingness to pay only when consumers regard it to be plausible. As previously discussed, the price of a focal product was also considered to serve as an anchor for consumers to estimate their willingness to pay. In this experiment, the price of the focal product was also controlled.

## General Discussion

The objective of this research was to examine the crucial role of the IRP in consumers’ willingness to pay for a promotional product after the promotion has been terminated. There are several studies that have recently discussed the contextual factors of consumers’ evaluation of supplementary products after a promotion has ended. For example, the inferential process suggested by Raghubir (2004) and Kamins et al. (2009) assumes that consumers evaluating a free supplementary product would attribute the promotion to either the low cost of the supplementary product or the low quality of the focal product. According to this argument, offering a product for free devalues it and decreases consumers’ willingness to pay after the promotion has been terminated. However, Palmeira and Srivastava’s (2013) research makes the opposite argument; based on the anchoring point, they propose that offering the supplementary product for free would increase consumers’ willingness to pay after the promotion was terminated. They argued that when the supplementary product was offered for free, there was no direct price information for that product, leading consumers to use the price of the focal product as an anchor to estimate their willingness to pay for the supplementary product. When the supplementary product was offered for a discounted price, this direct price information about the supplementary product would be the only anchor used by consumers to estimate their willingness to pay.

Due to the conflict between Kamins et al. (2009) and Palmeira and Srivastava (2013), this research explains consumers’ contextual factors when estimating their willingness to pay for the supplementary product after the promotion ends. According to reference price theory (Winer, 1986; Briesch et al., 1997; Kopalle and Lindsey-Mullikin, 2003; Mazumdar et al., 2005), the IRP of the product is based on consumers’ memory of prior purchases. In addition, the IRP is not invariable; it can be integrated with other external information (e.g., ERP) and updated in consumers’ purchasing experiments. Thus, determining whether external price information was employed by consumers to integrate their original IRP into a new IRP was proposed as the key to explaining the phenomenon. The present study focused on consumers’ willingness to pay for the supplementary product after the conditional promotions (e.g., purchasing a focal product and receiving the supplementary product for free or at a discounted price). In the conditional promotion, the promotional price of the supplementary product was the typical external price information that can be used by consumers to integrate their original IRP into a new IRP. However, not all promotional prices are employed by consumers. Some promotional prices (e.g., free, extremely low prices) are considered to be implausible prices that would not occur in normal purchasing. Consumers considered that the implausible promotional price would only occur in this temporary promotion. When the promotion was terminated, consumers’ IRP for the supplementary product did not change. For example, the company Apple offers a pair of Beats headphones when consumers purchase a laptop in Apple’s back-to-school seasonal promotion. Although the headphones were offered for free, consumers would not integrate this price with their original IRP because it was an implausible price after the promotion ended. Thus, this promotion did not influence consumers’ IRP for the Beats headphones in the non-promotional period.

The two experiments in this study provide strong support for the role of the IRP in consumers’ estimates of their willingness to pay after the promotion ends. This work proposed that consumers use a multiple anchoring judgment to estimate their willingness to pay for a supplementary product regardless of the type of promotional offer. The IRP is not only the crucial anchor to estimate the willingness to pay but also the criterion with which to determine whether other price information is plausible. If a large gap exists between the other price information (e.g., the promotional price of a supplementary product or the original price of a supplementary product) and the IRP of a supplementary product, the other price information would not be employed as plausible price information (i.e., the contrast effect) that can be integrated into a new IRP. Otherwise, the assimilation effect would occur, and the consumers would regard the other price information as a plausible price. When estimating their willingness to pay for a supplementary product after the promotion, consumers only use plausible price information as anchors in the multiple anchoring judgments. Price information that is higher (lower) than the IRP has a positive (negative) effect on the willingness to pay.

### Impacts

This research contributes to the literature in several ways. Consistent with Palmeira and Srivastava (2013), this study proposes that the well-documented anchoring effect has a crucial role in consumers’ evaluation of the price of a product in the absence of any price information (Tversky and Kahneman, 1974). Palmeira and Srivastava (2013) argued that consumers use different anchors for free supplementary products versus discounted supplementary products. When the supplementary product is free, consumers use the price of the focal product as an anchor to estimate the product’s value. Conversely, when the supplementary product is offered for a discounted price, consumers use the promotional price of the supplementary product as the anchor. This research improved the study of the anchoring evaluation from Palmeira and Srivastava (2013), because it examined the crucial role of the IRP in the multiple anchoring evaluations to explain consumers’ estimation of their willingness to pay.

Second, this research expands previous studies of reference price. Rajendran and Tellis (1994) defined the IRP as a type of reference price derived from previous prices paid or observed for a product. Other information about price (e.g., the price of the focal product, the promotional price of the supplementary product, and the original price of the supplementary product) can be regarded as types of ERPs or contextual prices (Rajendran and Tellis, 1994). Although ample research discusses the factors that influence reference price and the effect of reference price on consumers’ evaluations, the underlying process of how consumers evaluate a product when they face both IRPs and ERPs is ambiguous. This research discusses how the IRP and other external price information jointly serve as anchors to influence a consumer’s willingness to pay.

Third, this research examines the contextual factors of consumers’ evaluation of the plausibility of external price information (e.g., the price of a focal product, the promotional price of a supplementary product, or the original price of a supplementary product) in the short-term promotion. According to assimilation-contrast theory (Sherif and Hovland, 1961), consumers consider their distribution of prices to be acceptable. A product’s price information would be assimilated and would regulate a consumer’s evaluation only if the observed price falls within this distribution (Mazumdar et al., 2005). Thus, this study suggests that external price information can serve as a regulating factor of consumers’ willingness to pay only when the price information is considered to be plausible. Otherwise, this price information will generate a contrasting effect and will not be used by consumers to integrate their IRP and would not influence their willingness to pay for a product.

This research has several managerial implications. First, marketers should pay attention to the promotional strategy of product collocation. Products with a discounted price lead to a lower willingness to pay than products offered for free. Thus, if marketers do not want to devalue the product, offering it for free is a better idea than offering it at a discounted price. Furthermore, when setting a promotional price or an original price for a supplementary product, marketers should consider the reasonableness of the price; they should consider the relation between the actual price and the IRP. Hence, using a reasonable higher original price can increase consumers’ willingness to pay for the product after the promotion. This research provides a guideline to help marketers avoid the unintended devaluation of products by short-term incentives.

### Limitations and Future Research

This study has several limitations and highlights avenues for future research. First, consumers’ IRP was employed as a criterion to assess the plausibility of external price information. However, measuring the distribution of consumers’ IRP is difficult (Kalwani and Yim, 1992; Kalyanaram and Little, 1994; Han et al., 2001). The gap between the IRP and external price information was applied in this study to manipulate the plausibility of external price information, although this may not be the most accurate method. In addition, the certainty or confidence of consumers in their IRP may explain the impact of temporary promotional prices. An IRP with strong certainty or characterized by a narrow distribution (e.g., the price of a can of Coke in the supermarket) may be less likely to be influenced by promotional prices. Future research can identify additional anchors for consumers to use when estimating their willingness to pay and examine the relationship between these new types of price information and the IRP. In addition, the sample we used in the present research was comprised of university students. Participants from different departments were assigned to different conditions randomly. In order to decrease the bias of the purchasing experience between the student sample and general sample, we chose headphones, vacuum cups, and mugs as products in this study. Students are familiar with these three types of products, and have had past purchasing experience for them. However, the sampling method still has some potential impact on managerial application of this research. Since the student population cannot represent the population at large, it is impossible to generalize the results to general population. Thus, marketers need to pay attention to the potential impact of the student sample when applying the results of this research. Nevertheless, the present research can still provide valid insights into a cohort that represent a significant target market (i.e., student market) for companies of such products.

The social judgment theory may be another alternative explanation for this effect. According to this theory, people’s judgment of the various alternatives is spread across three latitudes: acceptance, neutrality (non-commitment), and rejection (Sherif et al., 1965). In the present research, we propose that the contextual price information can be considered as indicative of the plausible price, becoming an anchor only when the contextual price information is within the latitude of acceptance. If considered implausible, contextual price information cannot be used as an anchor. However, another alternative explanation is that consumers do not bring contextual price information into their IRP because the price is only within their latitude of neutrality, but not both neutrality and rejection. In other words, contextual price information which is within consumers’ latitude of rejection may also influence their IRP. This alternative explanation can be studied in future. More extreme prices (e.g., $0.01 for a car, or $1000 for a normal pen) could be used to ensure the prices fall within consumers’ latitude of rejection.

The specificity of the prices may also influence the consumer’s willingness to pay for the supplementary product. Odd pricing is a good example. Pricing products at one dollar below a whole number changing the leftmost digit to a lower level (e.g., $399 vs. $400) can have a significant effect on price perception, but this is not if the leftmost digit remains unchanged (e.g., $310 to $309) (Thomas and Morwitz, 2005). Thus, whether the specificity of the prices has a significant effect on consumer evaluations of the plausibility of the promotional price ($399 is plausible, but $400 is implausible) could be another avenue for future research.

## Ethics Statement

This study was carried out in accordance with the recommendations of National Central University Research committee with written informed consent from all subjects. All subjects gave written informed consent in accordance with the Declaration of Helsinki.

## Author Contributions

C-HL contributed to the improvement of the initial idea. MC designed the study and collected the data. Both two authors conducted the analysis, worked on the first draft and revised the conceptualizations and manuscript altogether. Both authors approved the final version of the manuscript.

## Conflict of Interest Statement

The authors declare that the research was conducted in the absence of any commercial or financial relationships that could be construed as a potential conflict of interest.
